# Adaptation strategies of giant viruses to low-temperature marine ecosystems

**DOI:** 10.1093/ismejo/wrae162

**Published:** 2024-08-23

**Authors:** Marianne Buscaglia, José Luis Iriarte, Frederik Schulz, Beatriz Díez

**Affiliations:** Faculty of Biological Sciences, Pontificia Universidad Católica de Chile, Av. Libertador Bernardo O’Higgins 340, Santiago 8331150, Chile; DOE Joint Genome Institute, Lawrence Berkeley National Laboratory, 1 Cyclotron Rd, Berkeley, CA 94720, United States; Millennium Institute Center for Genome Regulation (CGR), Av. Libertador Bernardo O’Higgins 340, Santiago 8331150, Chile; Center for Climate and Resilience Research (CR)2, Universidad de Chile, Av. Blanco Encalada 2002, Santiago 8370449, Chile; Centro de Investigación Dinámica de Ecosistemas Marinos de Altas Latitudes (IDEAL), Universidad Austral de Chile, Avda. El Bosque 01789, Punta Arenas 6210445, Chile; Instituto de Acuicultura y Medio Ambiente, Universidad Austral de Chile, Los Pinos s/n Balneario Pelluco, Puerto Montt 5500000, Chile; DOE Joint Genome Institute, Lawrence Berkeley National Laboratory, 1 Cyclotron Rd, Berkeley, CA 94720, United States; Faculty of Biological Sciences, Pontificia Universidad Católica de Chile, Av. Libertador Bernardo O’Higgins 340, Santiago 8331150, Chile; Millennium Institute Center for Genome Regulation (CGR), Av. Libertador Bernardo O’Higgins 340, Santiago 8331150, Chile; Center for Climate and Resilience Research (CR)2, Universidad de Chile, Av. Blanco Encalada 2002, Santiago 8370449, Chile

**Keywords:** giant viruses, NCLDV, cold adaptation, marine cold environments

## Abstract

Microbes in marine ecosystems have evolved their gene content to thrive successfully in the cold. Although this process has been reasonably well studied in bacteria and selected eukaryotes, less is known about the impact of cold environments on the genomes of viruses that infect eukaryotes. Here, we analyzed cold adaptations in giant viruses (*Nucleocytoviricota* and *Mirusviricota*) from austral marine environments and compared them with their Arctic and temperate counterparts. We recovered giant virus metagenome-assembled genomes (98 *Nucleocytoviricota* and 12 *Mirusviricota* MAGs) from 61 newly sequenced metagenomes and metaviromes from sub-Antarctic Patagonian fjords and Antarctic seawater samples. When analyzing our data set alongside Antarctic and Arctic giant viruses MAGs already deposited in the Global Ocean Eukaryotic Viral database, we found that Antarctic and Arctic giant viruses predominantly inhabit sub-10°C environments, featuring a high proportion of unique phylotypes in each ecosystem. In contrast, giant viruses in Patagonian fjords were subject to broader temperature ranges and showed a lower degree of endemicity. However, despite differences in their distribution, giant viruses inhabiting low-temperature marine ecosystems evolved genomic cold-adaptation strategies that led to changes in genetic functions and amino acid frequencies that ultimately affect both gene content and protein structure. Such changes seem to be absent in their mesophilic counterparts. The uniqueness of these cold-adapted marine giant viruses may now be threatened by climate change, leading to a potential reduction in their biodiversity.

## Introduction

Nucleocytoplasmic large DNA viruses (NCLDVs), also known as “giant viruses”, constitute the viral phylum *Nucleocytoviricota*. These viruses are characterized by large virion and genome sizes [1]. In marine environments, giant viruses are key players in the microbial food web as they infect eukaryotic microorganisms, from heterotrophic protists to diverse algal lineages [2–4]. Giant viruses impact host populations in manifold ways; through their role in the termination of large algal blooms [5, 6] and likely also through virus-induced metabolic host reprogramming [3, 7]. Giant virus diversity has been proposed to exceed that of bacteria and archaea [8]. Recently, a new clade of large eukaryotic DNA viruses named *Mirusviricota* has been proposed to be prevalent and diverse in the oceans [9]. Whereas giant viruses are ubiquitous in marine environments, the cold waters of the Arctic Ocean have been suggested as a hotspot for giant viruses, harboring a high number of unique phylotypes compared with other oceanic regions [10]. In contrast, the current information about giant viruses in the Southern Hemisphere cold marine environments is still scarce, as these ecosystems remain underexplored.

Temperature is known to play a crucial role in shaping microbial communities and is a significant selective pressure on marine microorganisms [11, 12]. To thrive in low-temperature environments, eukaryotes, bacteria, and archaea have evolved molecular and physiological adaptations to overcome the severe physicochemical constraints on cell function caused by low temperatures [13]. As low temperatures reduce reaction rates and tend to increase protein compactness, multiple mechanisms have evolved to increase protein flexibility, including changes in amino acid frequencies to reduce hydrogen bonds and salt bridges [13–15]. Other strategies involve encoding multiple copies of chaperones to cope with protein folding, the evolution of cold shock and antifreeze proteins, as well as the modification of biological membranes to increase fluidity through the accumulation of polyunsaturated fatty acyl chains [16]. Furthermore, the exploration of cold adaptations in bacteriophages from the Southern Ocean has also revealed the presence of several proteins related to cold-shock responses under positive selection, along with amino acid frequency patterns that enable them to thrive in cold temperatures [17]. Even though the effects of cold adaptation on viral genomes have been studied in bacteriophages, not much is known about how the much larger genomes of giant viruses have been impacted in low-temperature marine ecosystems.

Here, we employed genome-resolved metagenomics to better understand the distribution and cold-adaptive strategies of giant viruses (both *Nucleocytoviricota* and *Mirusviricota*) inhabiting austral ecosystems (Antarctica and Patagonian fjords) and compare our results to giant viruses from Boreal (Arctic) and temperate marine ecosystems. To achieve this goal, we first generated a catalog of giant virus metagenome-assembled genomes (GVMAGs) from austral cold marine environments combined it with previously published GVMAGs from the Arctic and Antarctica, and then analyzed their distribution across the oceans. We then analyzed their genomic composition and the molecular properties of their proteins and compared these to genomes of mesophilic giant viruses to identify and extend information on possible cold-adaptive capabilities that may allow these viruses to thrive in such extreme, low-temperature marine environments.

## Materials and methods

### Sample collection, processing, and sequencing

Chilean Patagonia seawater samples were collected during the austral spring of 2019, encompassing the fjords region located between 48° 5.281’S – 53° 34.972’S and 70° 36.349’W – 76° 0.9991’W. Antarctic seawater samples were collected at Chile Bay, Greenwich Island (62° 27.633’S; 59° 40.6’W), Southern Shetland Islands, during the austral summers of 2016 to 2020, and at South Bay, Doumer Island (64° 52.018’S; 63° 33.776’W) during the austral summer of 2020. During each sampling period, temperature was measured using a CTD-profiler (Sea-bird SBE19 plus) (Table S1).

To concentrate giant viruses present in the pico-size fraction (3–0.2 μm), 20 L of seawater (5–36 m) was collected in Niskin bottles and filtered through 200 and 20 μm polyester net, 3 μm polycarbonate filters, and finally 0.22 μm PES sterivex filters (Millipore) using a Cole Palmer System model no. 7553–70 peristaltic pump (6–600 rpm; pressure up to 2 bar). All filters were subsequently preserved in RNAlater buffer and maintained at −80°C until nucleic acid extraction and subsequent sequencing. The DNA concentrated on 0.22 μm PES filters was extracted following modified protocols previously described [18]. The 0.22 μm Sterivex filters were resuspended in xanthogenate buffer (1% potassium ethyl xanthogenate (Sigma-Aldrich, USA), 100 mM Tris–HCl (pH 7.4), 20 mM EDTA (pH 8), 800 mM ammonium acetate) with 1% SDS. The mixture was then incubated for 2 h at 65°C and hand-shaken every 30 min. Next, incubation tubes were placed on ice for 30 min. DNA extraction was performed with phenol-chloroform-isoamyl alcohol (25:24:1), and the residual phenol was further removed with chloroform-isoamyl alcohol (24:1). DNA precipitation and clean-up was fulfilled by overnight precipitation with cold isopropanol (−20°C) and successive washing with 70% ethanol. DNA quantification was measured with a Qubit 2.0 Fluorometer (Thermo Fisher Scientific, USA). DNA quality was assessed by spectrophotometry (A260/A280 ratio), and DNA integrity was confirmed by standard 1% agarose gel electrophoresis.

Viruses of the femto-size fraction (< 0.2 μm) were concentrated as previously described [19]. Briefly, 0.22 μm filtered water (20 L) was treated with 10 g/L FeCl_3_, then incubated for 1 h at room temperature, and then the FeCl_3_-treated water was filtered through a 1.0 μm polycarbonate membrane, which retained the flocculated viruses. Viruses concentrated on 1.0 μm polycarbonate membranes were resuspended in 0.1 M EDTA-0.2 M MgCl_2_–0.2 M ascorbic acid buffer pH 6.5, overnight at 4°C in the dark. To avoid cellular contamination, each resuspended fraction was re-filtered through 0.22 μm PES sterivex filters, and a DNase treatment was used to remove the remaining free DNA using 300 U of DNAse I per 1 ml of sample. DNAse I was inactivated by adding a final concentration of 100 mM EDTA / 100 mM EGTA, and the viruses were concentrated to 500 μl using Amicon MWCO 100 kDa ultracentrifugal filters. Viral DNA was then extracted using Wizard columns (Wizard DNA Purification Resin (Promega #A7181) and Wizard Mini Columns [Promega # A7211]), and DNA was quantified using the Qubit 2.0 Fluorometer (Thermo Fisher Scientific, USA).

DNA obtained from the 3–0.2 μm and < 0.2 μm fractions of each sample was sequenced using NovaSeq technology (Illumina; Roy J. Carver Biotechnology Center, Illinois) to obtain 27 metagenomes and 29 metaviromes, respectively (Table S1). In addition, five metagenomes were obtained from Antarctic samples of fractions between 150–0.2 μm. Shotgun genomic libraries were prepared with the Hyper Library construction kit from Kapa Biosystems (Roche) and sequenced on an S4 lane for 151 cycles from both ends of the fragments on a NovaSeq 6000 platform. Fastq files were generated and demultiplexed with the bcl2fastq v2.20 Conversion Software (Illumina). The quality of raw metagenomic reads was assessed using FastQC [20]. Quality trimming of sequences and adapters was performed, and hard trimming of the first 5–9 bases of 5′ (depending on the sample) and the last five bases of 3′ was applied to both pairs. The 3′ ends of both pairs were trimmed quality filtered (>25, >70 bp). Reads with ambiguous bases (N’s) and low complexity sequences were removed by PRINSEQ (−ns_max_p 0 -lc_method dust -lc_threshold 7) [21]. Paired-end quality-filtered reads were assembled using MEGAHIT [22]. The sequencing and assembly statistics of metagenomes and metaviromes are summarized in Table S1.

### Binning and taxonomic profiling of Antarctic and Patagonian GVMAGs

For each metagenome and metavirome, contigs larger than 5 kb were binned with MetaBAT (v.2) [23], and proteins of each bin were predicted with Prodigal [24], selecting the genetic code (1, 4, 6, or 11) that produced the highest coding density (percentage of bp predicted as part of a coding sequence in the MAG). To identify *Nucleocytoviricota* MAGs, proteins from each MAG were analyzed with hmmsearch (http://hmmer.org) against seven hidden Markov models (HMMs) built with seven *Nucleocytoviricota* orthologous groups (GVOGs) (SFII, RNAPL, PolB, TFIIB, TopoII, A32, and VLTF3) [25], and MAGs with more than one GVOG were detected (E-value cut-off of 1e-10) and selected for further analyses. GVOGs from each selected MAG were extracted and aligned using mafft (v.7) [26], and columns with less than 10% sequence information were removed from the alignment with trimAl (v.1.2) [27]. The alignment was used to perform a phylogenetic tree with 1384 reference sequences of *Nucleocytoviricota*, 16 eukaryotes, 18 bacteria, 12 archaea, and 110 *Mirusviricota* [9] using IQ-TREE (v.2.0.3) [28] and visualized with iTOL (v.6) [29]. Multiple maximum likelihood phylogenetic reconstructions were performed, removing MAGs that did not group with *Nucleocytoviricota* references. To identify *Mirusviricota* MAGs, MAGs with at least one hit against HMMs built with 5 *Mirusviricota* orthologous groups (HK97-fold MCP, Triplex1, Triplex2, Maturation, and Portal) [30] or against the curated HMMs of RNAPL, RNAPS, PolB, and TFIIS used to first describe *Mirusviricota* [9], were selected for further analysis (E-value cut-off of 1e-10). After generating a phylogenetic tree as described for *Nucleocytoviricota* MAGs, MAGs that did not group with *Mirusviricota* were removed from the analysis. The final *Nucleocytoviricota* and *Mirusviricota* MAGs (GVMAGs) phylogenetic trees were constructed based on a concatenation of seven marker genes (SFII, RNAPL, PolB, TFIIB, TopoII, A32, and VLTF3) [25], or four marker genes (RNAPL, RNAPS, PolB, TFIIS) [9], respectively, with 1000 bootstrap replicates. GVMAGs were dereplicated to obtain species-level phylotypes using standard thresholds of 95% average nucleotide identity over 85% alignment fraction [31]. The taxonomic affiliation of each GVMAG was determined based on the nearest reference neighbor. Genomic features and taxonomic affiliation of each GVMAG are summarized in Table S2. All GVMAGs presented a low copy number of cellular markers (using 56 HMMs considered as cellular marker proteins [32]), indicating low contamination.

To estimate how well giant viruses were sampled, sample-size-based rarefaction/extrapolation and coverage-based rarefaction/extrapolation curves were performed (Fig. S1) using the iNEXT package [33].

### Classifying Arctic, Antarctic, and Patagonian GVMAGs temperature distribution

The Antarctic and Patagonian GVMAGs generated in this study, as well as Arctic and Antarctic GVMAGs publicly available [9], were detected in 241 pico-size fraction metagenomes obtained from seawater samples ranging in temperature from −1.4°C to 30°C. This metagenomic dataset includes the metagenomes from the TARA Oceans dataset (Table S3). Read mapping was performed with Bowtie2 [34] using the “--end-to-end” and “--very-sensitive (-D 20 -R 3 -N 0 -L 20 -i S,1,0.50)” flags. A GVMAG with an average read depth of 2X along 70% of the MAG length was considered present in a sample, otherwise it was treated as zero (absent). To avoid false negatives, we only included in this analysis the GVMAGs obtained from the pico-size fraction. For publicly available GVMAGs obtained from co-assemblies, we selected those with a metagenomic signal >70% in the 5–0.2 μm size fraction [9] (Fig. S2).

### Cold adaptations in giant viruses

Proteins of GVMAGs from temperate waters (Atlantic, Indian, Mediterranean, and Pacific Oceans), as well as those from Arctic, Antarctic, and Patagonian waters either publicly available [9] or generated by this study, were predicted with Prodigal [24], selecting the genetic code (1, 4, 6, or 11) that produced the highest coding density (percentage of bp predicted as part of a coding sequence in the MAG). In this analysis, all GVMAGs were considered regardless of their fraction-size origin (Fig. S2C and S2F), as this should not, *a priori*, affect their possible adaptation to cold. To identify changes in amino acid frequencies or properties (such as aliphatic index, Grand Average of Hydropathy (GRAVY), and contents of acidic, charged, and polar uncharged residues) in proteins from cold environments compared to those from temperate environments, we used Orthofinder (v.2.5.4; [35]) to generate orthogroups and obtained 12 253 and 2310 orthogroups from *Nucleocytoviricota* MAGs and *Mirusviricota* MAGs, respectively. From these, we selected orthogroups (1050 of *Nucleocytoviricota* MAGs and 344 of *Mirusviricota* MAGs) containing one or more proteins from each marine ecosystem (Antarctic, Arctic, Patagonia, and Temperate) (Fig. S5), which were analyzed with in-house Python scripts based on Biopython modules Bio.SeqIO and Bio.SeqUtils.ProtParam [36]. A non-parametric analysis, the Mann–Whitney U-test, was used to test the difference between the distribution of each amino acid frequency or each molecular property measured in giant viruses proteins from cold ecosystems (Antarctic, Arctic, or Patagonia) and those from temperate waters. Normal distribution was priorly tested using the Shapiro–Wilk test (*P* value <0.05).

Additionally, the predicted proteins were annotated with the KOfam database and KofamScan [37] (E-value cut-off of 1e10^5^). Both, cold-exclusive and cold-overrepresented KEGG Orthologs (KOs) were selected based on a pool of KEGG-annotated proteins from cold and temperate waters. Cold overrepresentation was considered by calculating the prevalence of the KO in the genomes of cold or temperate environments, and those that were at least 3 times more prevalent in the cold environments were selected. To avoid considering KOs randomly present in cold or temperate environments, only KOs detected in two or more GVMAGs per environment were considered for the analysis.

## Results

### Enriching the giant viruses dataset of cold austral marine ecosystems

**Fig. 1:**
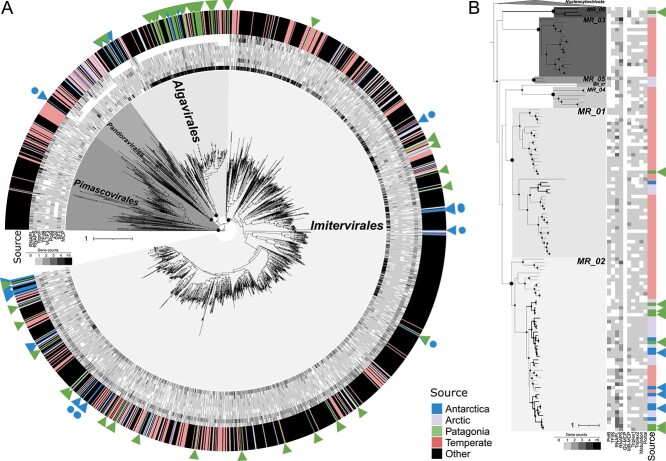
**Taxonomic affiliations of marine giant viruses.** (A) Maximum-likelihood phylogenetic tree of the *Nucleocytoviricota* based on a concatenated protein alignment of SFII, RNAPL, PolB, TFIIB, TopoII, A32, and VLTF3. From the center outward, the first nine rings represent the copy number of each of these *Nucleocytoviricota* marker genes plus MCP and RNAPS, followed by the source of each *Nucleocytoviricota* MAG, shown in different colors (“other” refers to giant viruses from the giant virus database [25] used as a phylogenetic framework for constructing the tree). (B) Maximum-likelihood phylogenetic tree of the *Mirusviricota* based on a concatenated protein alignment of PolB, TFIIS, RNAPL, and RNAPS. From left to right, the first ten rings represent the copy number of each marker gene, *Nucleocytoviricota* double jelly-roll MCP (GV_MCP), *Mirusviricota* HK97-fold MCP (MR_MCP), Triplex1, Triplex2, maturation, and portal genes, followed by the source of each *Mirusviricota* MAG, shown in different colors. GVMAGs generated in this study are indicated with a triangle. GVMAGs representing families or genera not previously detected in Antarctic waters are shown with dots. Black dots inside the trees indicate bootstrap support from ML (1000 bootstrap replicates) >95%, highlighting those supporting Order level clades. The scale bar represents one substitution per site. *Pokkesviricetes* is collapsed in the *Nucleocytoviricota* tree.

Global metagenomic studies have explored marine giant viruses communities using samples from diverse oceanic regions, primarily through the TARA Oceans project [38]. However, giant virus research in polar regions has primarily relied on samples from the Arctic Ocean comprising 34 metagenomes of the pico-size fraction (0.2–3 μm), 34 of the femto-size fraction (< 0.2 μm), and a co-assembly of 108 metagenomes (0.8 μm–2 mm). In contrast, in the Southern Hemisphere, only seven samples from Antarctica were available, and regions such as the Patagonian fjords remained entirely unexplored. To broaden our understanding of giant viruses in cold austral marine environments, we collected and sequenced marine samples from both Antarctica and the sub-Antarctic ecosystem located in the Chilean Patagonia, obtaining 32 metagenomes (0.2–3 μm and 0.2–150 μm) and 29 metaviromes (< 0.2 μm) (Table S1) that led to the recovery of 31 Antarctic and 67 Patagonian *Nucleocytoviricota* MAGs, and four Antarctic and eight Patagonian *Mirusviricota* MAGs (all dereplicated at 95% ANI) (Fig. 1). All GVMAGs were detected in the metagenomes, and despite 25.5% of the *Nucleocytoviricota* MAGs being assembled from metaviromes, no phylotypes (species-rank level) were exclusively detected in the femto-size fraction (Fig. S2A, B). In our phylogenetic analysis, the recovered giant viruses grouped the orders *Imitervirales*, *Algavirales*, *Pandoravirales*, and the proposed MR_01, MR_02, and MR_06 orders (Fig. 1 and Fig. S2B, E). The generation of these *Nucleocytoviricota* and *Mirusviricota* MAGs expands the existing TARA Antarctic dataset deposited in the GOEV database almost threefold and twofold, respectively (Fig. S2C, F). These new phylotypes provide evidence of genera and families present in these ecosystems that were not detected in previous studies (Fig. 1) [9]. Additionally, we report giant viruses from the underexplored sub-Antarctic cold ecosystems represented here by the Chilean Patagonia, which were mainly affiliated with *Algavirales*.

### Distribution patterns of giant viruses from cold environments at the global ocean scale

We investigated the distribution of cold environments GVMAGs in marine metagenomic samples at temperatures ranging from −1.4°C to 30°C, using Arctic and Antarctic TARA GVMAGs previously deposited in the GOEV database [9], in addition to our Antarctic and Patagonian GVMAGs (Fig. 1 and Fig. S2). To avoid potential false negatives linked to the sample size fractions analyzed, we performed the detection of pico-size fraction GVMAGs in the pico-size fraction metagenomes, as most of them are expected to be in the pico-size range. Given that GOEV TARA MAGs were generated from co-assemblies of fraction sizes between 0.8 μm and 2 mm, we considered only those with a metagenomic signal >70% in the pico-size-like fraction (0.2–5 μm) (Fig. S2) [9].

**Fig. 2:**
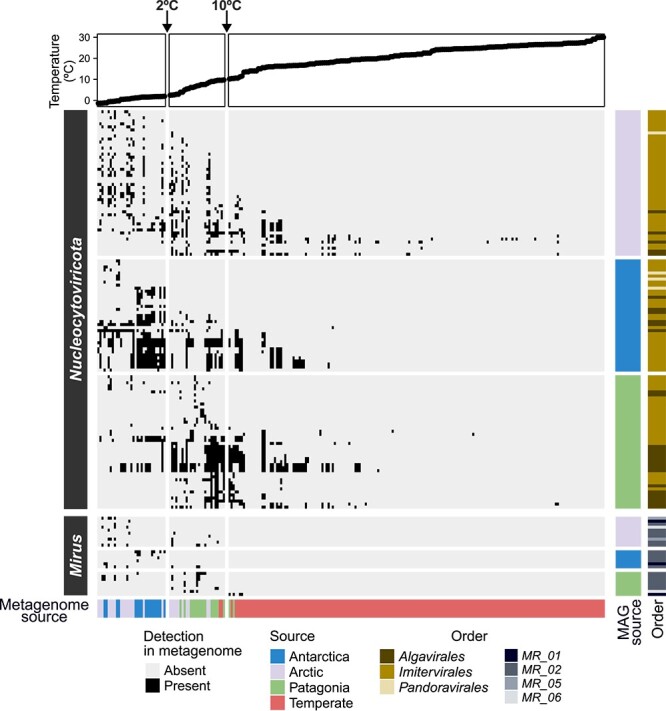
**Temperature- and geography-driven distribution of giant viruses from cold marine environments.** Distribution of *Nucleocytoviricota* or *Mirusviricota* MAGs from the Arctic (GOEV database), Antarctic (this study + GOEV database), and Patagonian fjords (this study). The X-axis corresponds to metagenomes, and the Y-axis corresponds to GVMAGs. The MAG source indicates where the GVMAG was obtained (Antarctic, Arctic, or Patagonian marine samples), whereas the metagenome source indicates where the metagenome was obtained (Antarctic, Arctic, Patagonian, or temperate marine samples). All GVMAGs were obtained either from the pico-size fraction (0.2–3 μm) or, when generated from co-assemblies, had a metagenomic signal of more than 70% in the pico-size-like fraction (0.2–5 μm) (summarized in Fig. S2) [9]. To analyze giant virus distribution, read mapping was conducted using pico-size metagenomes from this study and public databases, covering a temperature range of −1.4°C to 30°C. A GVMAG was considered present in a sample if it had an average read depth of 2X along at least 70% of the MAG length; otherwise, it was treated as absent.

Our analysis revealed a significant negative correlation between temperature and the number of GVMAGs detected, suggesting a temperature-driven limitation of their dispersal (Fig. S3A). Most Antarctic and Arctic *Nucleocytoviricota* MAGs were detected at temperatures below 2°C (100% and 87.5%, respectively) (Fig. 2 and Fig. S3B); however, the Antarctic ones, mostly affiliated with the proposed meso_4 subfamily in the order *Imitervirales*, were highly restricted to this temperature range (45.9%), in marked contrast to the Arctic *Nucleocytoviricota* MAGs (14.5%) (Fig. 2 and Fig. S3C). Overall, more than half of the Arctic (58.3%) and Antarctic *Nucleocytoviricota* MAGs (59.5%) were found only at temperatures below 10°C, with <10% being detected above 18°C (Fig. 2 and Fig. S3B, C). In contrast, a smaller proportion of Patagonian *Nucleocytoviricota* MAGs were found only at temperatures below 10°C (38.6%), and none were restricted to temperatures below 2°C (Fig. 2 and Fig. S3C).

In turn, the temperature distribution for Antarctic and Arctic *Mirusviricota* MAGs was even narrower than for *Nucleocytoviricota*, with all detected only below 10°C, and a high proportion, affiliated with the proposed orders MR_01, MR_02, MR_05, and MR_06, restricted to temperatures below 2°C (50% and 83.3%, respectively) (Fig. 2 and Fig. S3D, E). Conversely, most Patagonian *Mirusviricota* MAGs were detected only at temperatures below 10°C (87.5%), but none were restricted to temperatures below 2°C (Fig. 2 and Fig. S3E).

The analysis further revealed that a high percentage of *Nucleocytoviricota* MAGs from cold environments were exclusive to Antarctica and the Arctic (34.0% and 29.5%, respectively, Fig. S4A), with an even higher proportion found for *Mirusviricota* MAGs (55.6% Antarctic and 57.1% Arctic, respectively, Fig. S4B). This trend was not followed by Patagonian GVMAGs (16.9% of *Nucleocytoviricota* and 27.3% of *Mirusviricota*, Fig. S4A, B). Nevertheless, the ratio of unique/total pico-size *Nucleocytoviricota* MAGs calculated by sample was not significantly different between Antarctica and the Arctic, or between Antarctica and Patagonia (Fig. S4C). More data is needed for performing a more comprehensive analysis of *Mirusviricota*, as most of the samples with *Mirusviricota* MAGs contained only one or two phylotypes.

### Compositional changes in genomes of cold-adapted giant viruses

**Fig. 3:**
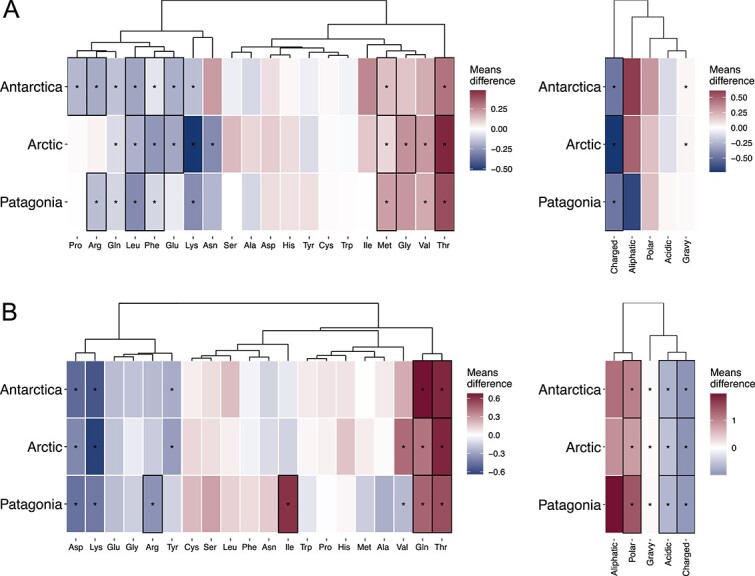
**Temperature-dependent amino acid composition in giant viruses from cold marine waters.** Average differences in amino acid frequencies and chemical properties of the proteins of *Nucleocytoviricota* (A) and *Mirusviricota* (B) MAGs from the Arctic, Antarctic, and Patagonian fjords compared to those from temperate waters. The analysis was conducted by comparing orthogroups (1050 for *Nucleocytoviricota* and 344 for *Mirusviricota*) common to both cold and temperate giant viruses. Amino acids and chemical properties with changes previously associated with cold adaptations are outlined in black boxes.

To investigate possible cold adaptations in giant viruses from all size fractions (summarized in Fig. S2), we initially focused on identifying changes in amino acid frequencies in their proteins compared to those of giant viruses exclusively detected in temperate waters (> 10°C) (344 *Nucleocytoviricota* MAGs and 76 *Mirusviricota* MAGs, Fig. 1) [9]. Our analysis, using common orthogroups of *Nucleocytoviricota* MAGs from cold and temperate waters (Fig. S5A), revealed significant differences in the average frequencies of six amino acids (threonine, methionine, lysine, leucine, glutamine, and phenylalanine) in the proteins of *Nucleocytoviricota* GVMAGs from all cold ecosystems compared to those from temperate ones (Fig. 3A). The increase in threonine and methionine, and the decrease in leucine and phenylalanine, are molecular adaptations previously reported in microorganisms to low temperatures [13–15, 17, 39–42]. When comparing specific variations within each cold ecosystem, the Antarctic *Nucleocytoviricota* MAGs exhibited the highest number of significant variations (seven amino acids) previously associated with cold adaptation. In turn, when comparing the amino acid frequencies of common orthogroups of *Mirusviricota* MAGs from cold and temperate waters (Fig. S5B), we detected significant differences in the average frequencies of four amino acids (aspartate, lysine, glutamine, and threonine) in proteins of *Mirusviricota* MAGs from all cold ecosystems compared to those from temperate ones (Fig. 3B), two of which had been previously reported in psychrophiles [15, 40, 41]. *Mirusviricota* MAGs from Antarctica and the Arctic shared most of their significant variations (five out of six amino acids), whereas those from Patagonia exhibited the highest number of previously reported molecular cold adaptations (four amino acids).

We analyzed the GRAVY, aliphatic index, and the content of acidic, charged, and polar uncharged residues. We found significantly lower levels of charged amino acids in GVMAGs proteins from all cold compared to temperate ecosystems, as well as a higher content of polar uncharged amino acids, and a lower content of acidic amino acids in proteins of *Mirusviricota* MAGs (Fig. 3A, B), all changes previously reported in psychrophiles [43]. Additionally, we found a significant change in the GRAVY index of proteins from Antarctic and Arctic *Nucleocytoviricota* MAGs, and in all *Mirusviricota* MAGs.

### Gene functional changes in cold-adapted giant viruses

**Fig. 4:**
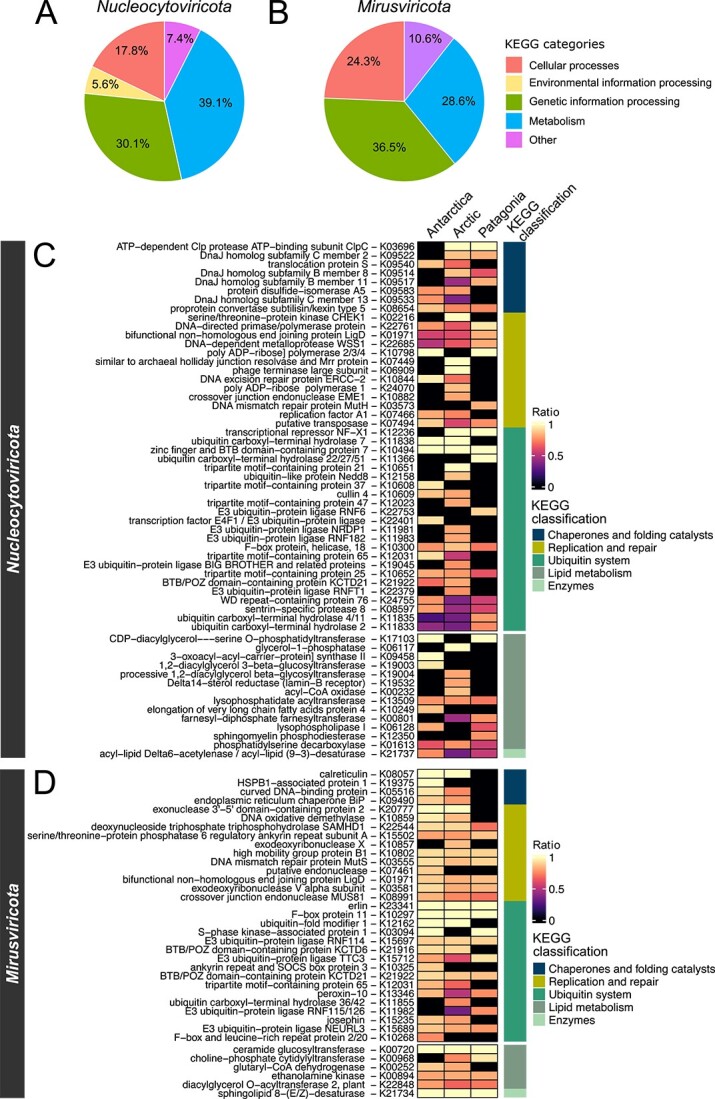
**Exclusive or overrepresented KEGG functions in giant viruses from cold marine waters.** (A, B) Main KEGG categories found exclusively or overrepresented in cold *Nucleocytoviricota* and *Mirusviricota* MAGs. (C, D) KOs classified in categories potentially associated with the cold adaptation of giant viruses. The ratio was calculated as the prevalence of the KO in cold environments GVMAGs divided by the sum of its prevalence in both cold and temperate environments GVMAGs. KOs that were at least three times more prevalent (0.75 < ratio < 1.0) in the cold than in temperate GVMAGs were classified as “cold-overrepresented”. KOs detected exclusively in cold environments GVMAGs were classified as “cold-exclusive” (ratio = 1.0).

To elucidate the genetic adaptations of giant viruses from Arctic, Antarctic, and Patagonian marine environments, we compared their KEGG-annotated proteins with those of giant viruses from temperate marine waters (shown in Fig. 1). Our analysis identified KEGG orthologs (KOs) that were either exclusive to or overrepresented (> 3 times more prevalent) in *Nucleocytoviricota* (82 and 215 KOs, respectively) and *Mirusviricota* (41 and 102 KOs, respectively) MAGs from cold environments compared to their temperate counterparts (Fig. S6, Table S4). These KO functions were mainly related to genetic information processing (30.1% and 36.5%, respectively) and metabolism (39.1% and 28.6%, respectively) (Figs. 4A, B). Among these KOs, we found genes involved in common cold-adaptive strategies of microorganisms. For example, a high proportion of the genetic information processing KOs were related to the ubiquitin system (24.7% and 23.2%, respectively), replication and repair processes (13.1% and 17.4%, respectively), and chaperones and folding catalysts (8.1% and 5.8%, respectively) (Fig. 4C, D; Table S4), which may be linked with cold temperatures placing severe constraints on protein folding and nucleic acid structures [13, 44]. Additionally, we found that about 10% of the metabolism KOs were related to lipid metabolism, including desaturases, elongation of very long chain fatty acids proteins, and proteins related to the diacylglycerol and sterol metabolism proteins (Fig. 4B, C), enzymes previously associated with lipid remodeling and linked to cold adaptation [13, 45–50].

To explore how these gene functional changes on giant viruses may impact their hosts, we searched for giant viruses encoding possible cold-related functions and phylogenetically close to a virus with a known host. We found an Antarctic GVMAG closely related to the *Phaeocystis globosa* virus; nevertheless, the overrepresented functions it encoded (ubiquinone biosynthesis protein [K03688] and regenerating islet-derived proteins 4 [K22244]) were not related to common cold adaptations.

## Discussion

Given the limited information about giant viruses in the austral (Antarctic and Patagonian fjords) marine environments, we expanded the current austral giant viruses dataset to determine their oceanic distribution and compare it with their Arctic counterparts. With this enriched polar dataset, we also obtained an estimation of giant viruses’ adaptations to the cold. The current scarcity of data in austral regions underscores the importance of our study, offering new insights into the viral ecology of cold environments.

### Antarctic and Patagonian giant viruses further expand the biogeography of *Nucleocytoviricota* and *Mirusviricota* in the oceans

Previous studies in the Arctic Ocean suggested it as a hotspot for giant viruses, with geographical barriers and environmental gradients contributing to the high proportion of unique giant virus phylotypes [10]. This trend was not observed in Antarctica, possibly due to the low number of samples from that environment [10]. In this study, we expanded the dataset of giant viruses by mining 61 new metagenomes and metaviromes from cold austral environments, which allowed us to determine that Antarctic marine waters also harbor a high proportion of unique giant virus phylotypes of *Nucleocytoviricota* (34.0%) and *Mirusviricota* (55.6%), similar to the proportion found in the Arctic (*Nucleocytoviricota*: 29.4%; *Mirusviricota*: 57.1%). The existence of a dispersal barrier for giant viruses was proposed in a recent Arctic study, likely associated with selective pressure from temperature [51]. In Antarctica, the Circumpolar Current forms an open-ocean dispersal barrier suggested to maintain the Antarctic waters and their microbial communities relatively isolated from the rest of the oceans [52–54]. We postulate that these environmental and geographical barriers may also shape Antarctic giant virus communities. However, it is important to consider that the number of samples, their processing, and environmental parameters during sampling, among other factors, may affect conclusions regarding the uniqueness of phylotypes present in these ecosystems.

Our Patagonian GVMAGs chiefly expanded the diversity of giant viruses, especially of *Algavirales*. More than half of the *Algavirales* GVMAGs representatives were assembled from the femto-size fraction; therefore, including metaviromes is particularly important when studying *Algavirales*-rich zones like Patagonia. Within the Patagonian coastal marine system, the interaction between oceanic waters and freshwater from glacial melting, river discharges, and precipitations generates strong vertical and horizontal gradients in salinity, nutrients, and light availability. These physicochemical gradients impact the pelagic microbial communities, leading to seasonal fluctuations in primary production, which notably peaks during spring and autumn [55, 56]. Our Patagonian samples, collected in spring, reached chlorophyll-*a* concentrations up to 4.3 mg m^−3^, which may explain the high proportion of *Algavirales* found in these samples, likely infecting photosynthetic planktonic hosts.

Our results showed that giant viruses from cold environments exhibit a limited distribution across the oceans, suggesting the existence of specific temperature-associated barriers to their dispersal. A significant proportion of giant virus phylotypes from Antarctic and Arctic ecosystems were only detected at temperatures below 2°C and 6°C, respectively, indicating that certain phylotypes are restricted to cold temperatures and to psychrophilic hosts (microbes indigenous to cold environments [57]). In contrast, most giant viruses from sub-Antarctic waters, represented in this study by the Chilean Patagonia fjords, were not restricted to these low temperatures. This suggests that, although the Patagonian fjord region constitutes a cold marine ecosystem with temperatures that can drop below 3°C, the even more extreme temperatures of Antarctic and Arctic waters create a stronger environmental barrier that hinders the dispersal of certain giant virus phylotypes. Consequently, if temperature indeed poses a significant environmental barrier to the dispersal of giant viruses, the expected rise in temperatures linked to climate change could potentially facilitate the dispersal of sub-polar viral lineages that remain undetected under current conditions. This could have unpredictable repercussions on the ecological balance of marine ecosystems in polar regions. Additionally, the high percentage of potentially unique giant virus phylotypes present in Antarctic and Arctic ecosystems could also be affected by environmental changes related to climate change, such as global warming, leading to a consequent loss in giant virus biodiversity.

### Genomic adaptations of giant viruses in low-temperature marine ecosystems

Microorganisms inhabiting cold environments have evolved physiological and molecular strategies to thrive at low temperatures [13, 58]. Here, we determined that giant viruses from the Arctic, Antarctica, and Chilean Patagonia exhibit changes in both the amino acid frequencies of their proteins and the gene content of their genomes compared to temperate giant viruses, changes that potentially enable them to thrive in cold environments. Considering that viruses rely on their hosts for replication, their distribution and fitness in the oceans are likely constrained by both their own sensitivity to environmental factors and that of their hosts. Therefore, we propose that the cold adaptations of giant viruses described here result from environmental constraints affecting virus-host co-evolutionary processes. Such processes include giant virus-encoded genes that lead to proteins with features enhancing host fitness under adverse environmental conditions, such as low temperatures, and that ensure efficient viral housekeeping such as transcription, genome maintenance, and viral packaging.

Regarding protein-level putative cold adaptations, proteins with higher contents of methionine, threonine, and polar uncharged amino acids, as well as lower contents of leucine, phenylalanine, charged, and acidic amino acids were detected in the GVMAGs from all cold ecosystems tested compared to temperate ones, changes previously observed in proteins of eukaryotes [42], bacteria [15, 39, 40], archaea [41, 59], and bacteriophages [17] in cold environments compared to their mesophilic counterparts. It has been suggested that the high entropy (degrees of freedom) and the lack of interacting groups associated with methionine residues allow greater flexibility in proteins [14]; and that the high conservation of four methionine residues provides additional flexibility to enzyme cleavage active sites [60]. Conversely, increased threonine residues may induce decreased stability of hydrogen bonds in the dynamic structure of cold-adapted enzymes [15]. Psychrophiles also tend to avoid leucine and phenylalanine as they stabilize helical structures [40, 61, 62]. A general reduction of charged residues (basic and acidic) has also been detected in psychrophilic proteins [41, 43], as they tend to form salt bridges and electrostatic interactions that correlate with higher overall protein stability. Moreover, charged amino acid content has been suggested to play a crucial role in modeling to discriminate psychrophilic from non-psychrophilic proteins [63]. Additionally, the overall higher content of uncharged polar amino acids may partly offset the overall reduction of charged residues [41, 43], which in our study seemed to be related to a positive shift in threonine and glutamine frequencies.

The higher glycine frequencies found in the Arctic *Nucleocytoviricota* proteins align with previous findings in cold-adapted microorganisms [13, 43], as glycine is recognized as a helix-breaker that could also provide greater conformational freedom due to its small size. Meanwhile, the lower asparagine frequencies in Arctic *Nucleocytoviricota* proteins have not been previously reported in psychrophiles but could prevent NH-π interactions and their stabilizing effect [64]. Proteins from Antarctic *Nucleocytoviricota* MAGs also showed lower proline contents, amino acid covalently bound to the nitrogen atom of the peptide group which imposes constraints on peptide backbone rotations and can contribute to the overall lower flexibility of proteins [13, 14, 39, 65, 66]. Additionally, the higher isoleucine and lower valine frequencies found in the Patagonian *Mirusviricota* proteins are consistent with valine-to-isoleucine substitutions previously reported in psychrophilic proteins [67].

Beyond molecular adaptations, microorganisms have developed physiological adaptations by acquiring or evolving genes/proteins that can counteract the unfavorable effects of living at low temperatures [13]. Giant viruses from cold environments, compared to their temperate counterparts, also appear to possess exclusive or overrepresented genes that have already been associated with cold adaptations in other organisms. For example, genes related to genetic information processing, such as ribonucleases, may have been maintained in giant viruses to facilitate the removal of misfolded mRNA [44, 68], potentially improving their fitness in these cold environments. The overrepresentation of chaperones and the ubiquitin system genes might also help in protein folding, a process suggested to be challenging at low temperatures [13, 69–71]. Desaturases, which catalyze the introduction of double bonds into fatty acid chains, were found exclusively or overrepresented in giant viruses from cold environments. These enzymes have been linked to cold adaptation in microorganisms as they can regulate membrane fluidity in response to low temperatures, generating a selective advantage to thrive in the cold [45–47, 49]. Recently, the unsaturated fatty acid biosynthesis pathway has been shown to be significantly enriched in Arctic giant viruses [51], and together, these findings emphasize the potential importance of this trait for giant viruses inhabiting the cold marine environments of the Arctic, Antarctic, and Patagonia. Additionally, genes related to diacylglycerol metabolism were found exclusively or overrepresented in all three cold environments. At low temperatures, cell membrane damage can result from diacylglycerol forming a destabilized hexagonal II (H_II_)-type phase with phosphatidic acid, and enzymes converting diacylglycerol to other molecules (such as triacylglycerol) have been shown to play a crucial role in cold tolerance [48]. We do not rule out the existence of other genes that were not identified here and that might be linked to cold adaptation in giant viruses; however, our findings suggest a potential selective pressure on these genes, which in turn allows viral reprogramming of host metabolism to improve their fitness in cold environments.

## Conclusion

Our study sheds light on the distribution of giant viruses in cold marine ecosystems of Antarctica, the Arctic, and the Patagonian fjords, and highlights the uniqueness of giant viruses in these environments. The presence of genes with potential roles in cold adaptation, together with molecular changes in their proteins, suggest that giant viruses have evolved adaptations to improve their fitness in these challenging environments.

## Supplementary Material

FigS1_aug_wrae162

FigS2_aug_wrae162

FigS3_aug_wrae162

FigS4_aug_wrae162

FigS5_aug_wrae162

FigS6_aug_wrae162

TableS1_aug_wrae162

TableS2_aug_wrae162

TableS3_aug_wrae162

TableS4_aug_wrae162

## Data Availability

The datasets and in-house Python scripts generated in this study are available online (Figshare repository 10.6084/m9.figshare.26314543). The metagenomic data generated in this study will be available in SRA upon publication.
