# Supplementary figures and images for: Adaptation strategies of giant viruses to low-temperature marine ecosystems
Source: ISME J. 2024 Aug 23;18(1):wrae162. doi: 10.1093/ismejo/wrae162 (PMC11512752; doi:10.1093/ismejo/wrae162)

**A**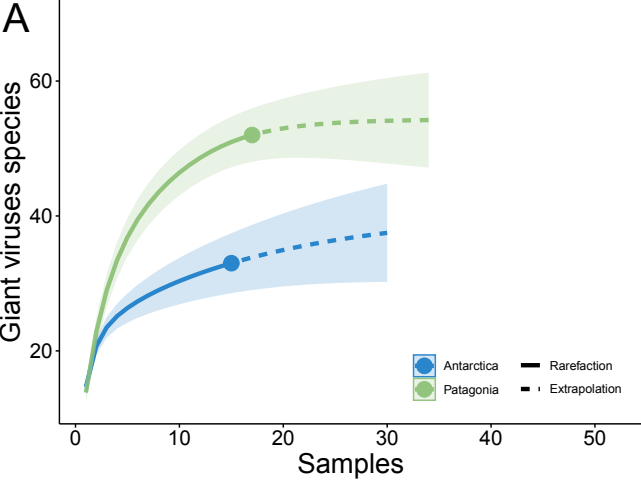**B**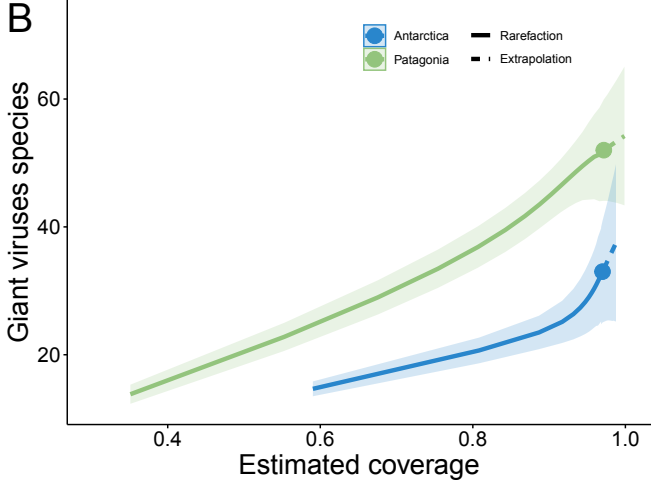

Supplement: FigS1_aug_wrae162 [file figs1_aug_wrae162.pdf]

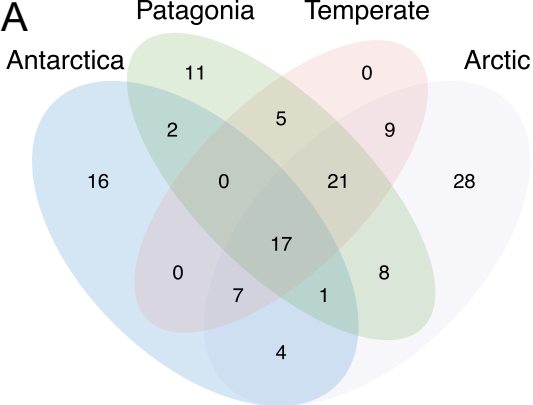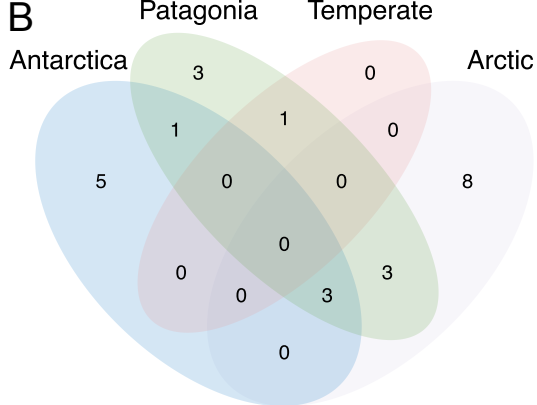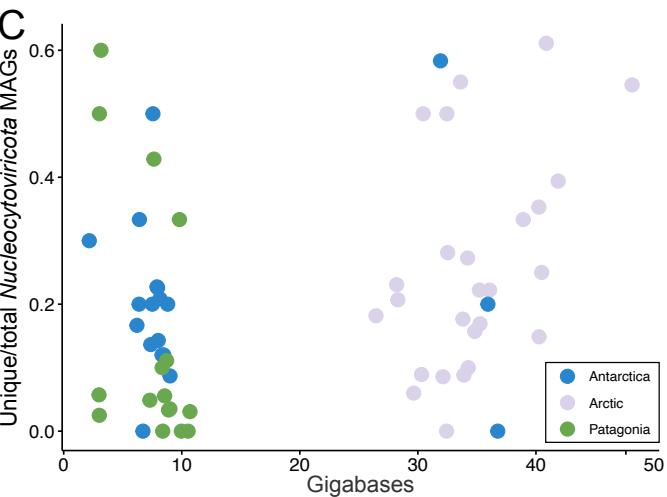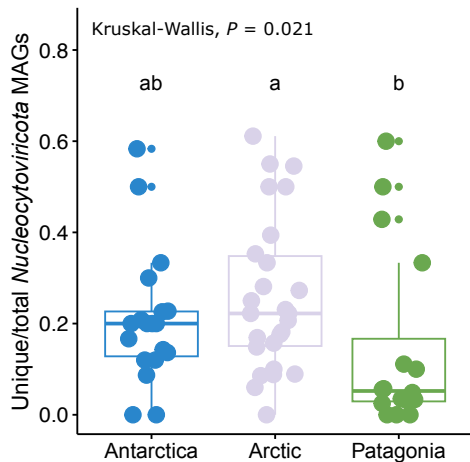

Supplement: FigS4_aug_wrae162 [file figs4_aug_wrae162.pdf]

**A**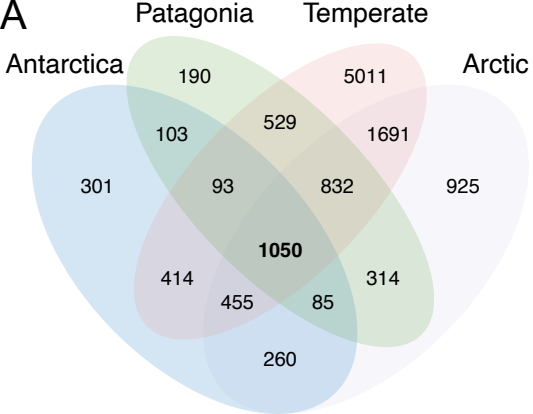**B**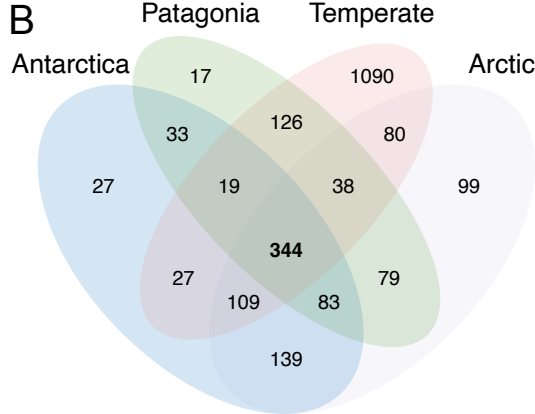

Supplement: FigS5_aug_wrae162 [file figs5_aug_wrae162.pdf]

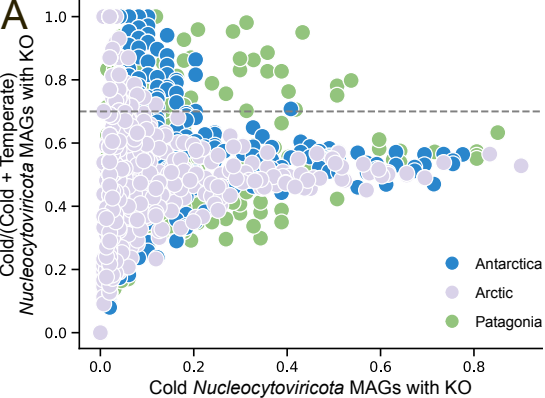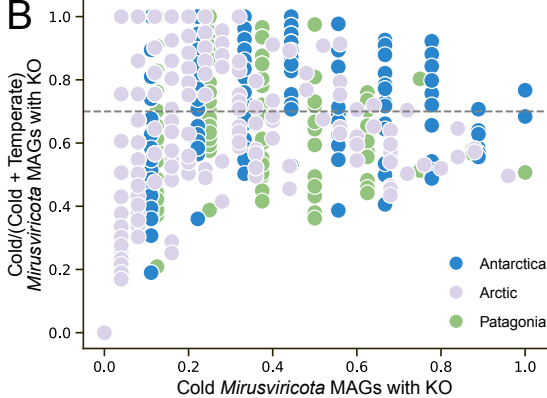

Supplement: FigS6_aug_wrae162 [file figs6_aug_wrae162.pdf]
