# Supplementary material for: Adaptation strategies of giant viruses to low-temperature marine ecosystems
Source: ISME J. 2024 Aug 23;18(1):wrae162. doi: 10.1093/ismejo/wrae162 (PMC11512752; doi:10.1093/ismejo/wrae162)

A

Nucleocytoviricota

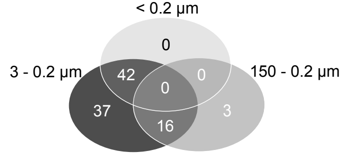

B

	Nucleocytoviricota			
	Order	150 - 0.2 µm	3 - 0.2 µm	< 0.2 µm
Antarctica	*Pandoravirales*	0	1	0
	Algavirales	1	4	2
	Imitervirales	5	18	0
Patagonia	*Pandoravirales*	0	0	0
	Algavirales	0	18	23
	Imitervirales	0	26	0

C

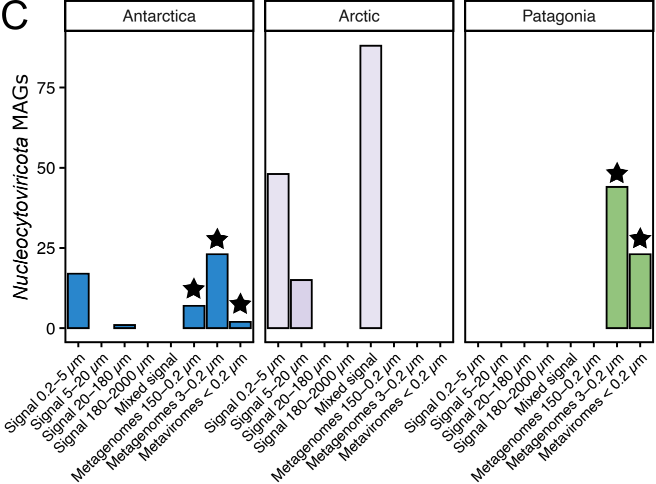

D

Mirusviricota

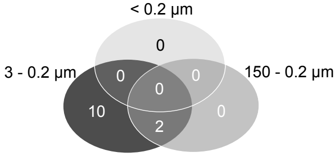

E

	Mirusviricota			
	Order	150 - 0.2 µm	3 - 0.2 µm	< 0.2 µm
Antarctica	MR_01	0	0	0
	MR_02	2	2	0
	MR_06	0	0	0
Patagonia	MR_01	0	1	0
	MR_02	0	6	0
	MR_06	0	1	0

F

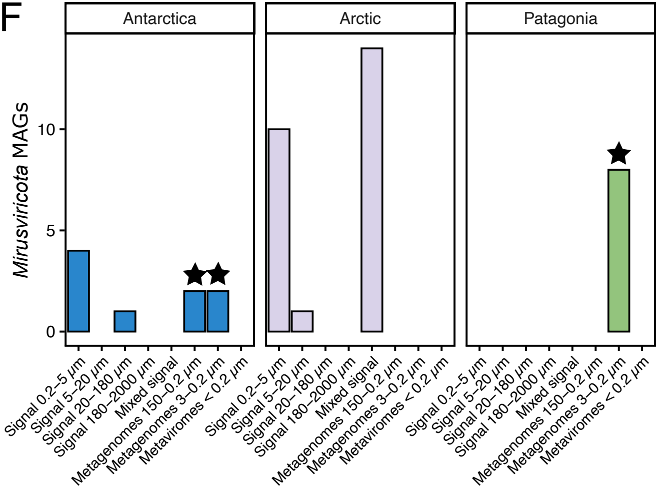

Supplement: FigS2_aug_wrae162 [file figs2_aug_wrae162.pdf]
