# Supplementary material for: Adaptation strategies of giant viruses to low-temperature marine ecosystems
Source: ISME J. 2024 Aug 23;18(1):wrae162. doi: 10.1093/ismejo/wrae162 (PMC11512752; doi:10.1093/ismejo/wrae162)

A

	Nucleocytoviricota		*Mirusviricota*	
	Spearman's rho	*P* value	Spearman's rho	*P* value
Antarctica	-0.73	2.57e-06	-0.67	2.7e-05
Arctic	-0.68	1.79e-05	-0.64	8.1e-05
Patagonia	-0.61	2.00e-04	-0.71	6.2e-06

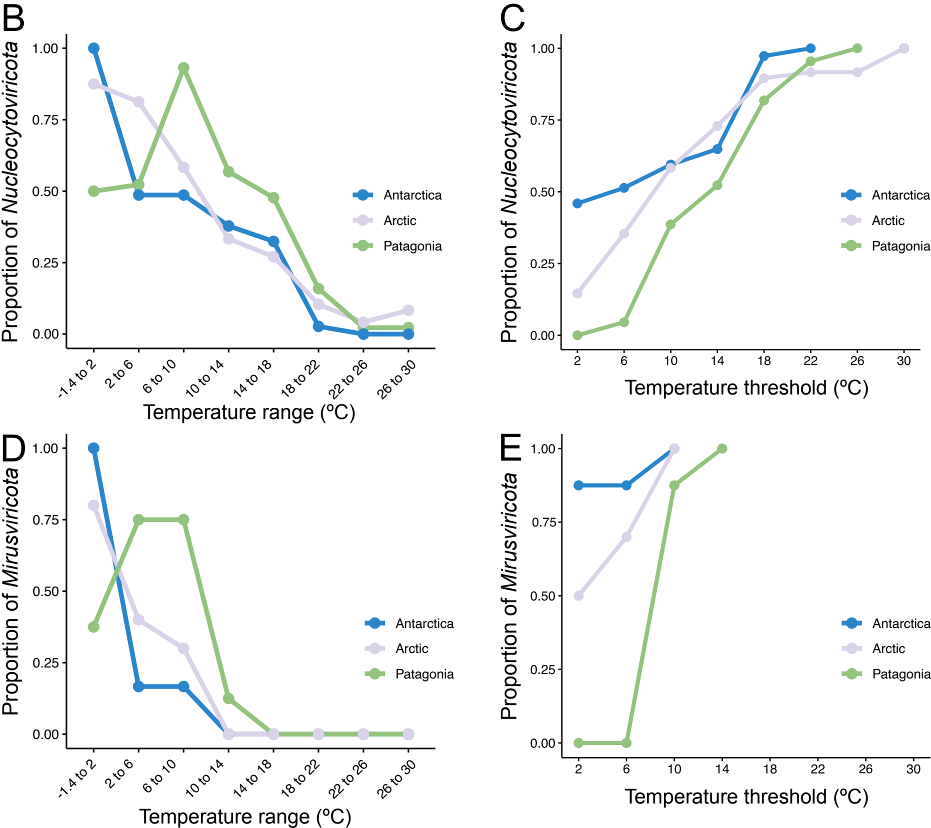

Supplement: FigS3_aug_wrae162 [file figs3_aug_wrae162.pdf]
